# SARS-CoV-2 Seropositivity in Urban Population of Wild Fallow Deer, Dublin, Ireland, 2020–2022

**DOI:** 10.3201/eid3008.231056

**Published:** 2024-08

**Authors:** Kevin Purves, Hannah Brown, Ruth Haverty, Andrew Ryan, Laura L. Griffin, Janet McCormack, Sophie O’Reilly, Patrick W. Mallon, Virginie Gautier, Joseph P. Cassidy, Aurelie Fabre, Michael J. Carr, Gabriel Gonzalez, Simone Ciuti, Nicola F. Fletcher

**Affiliations:** University College Dublin, Dublin, Ireland (K. Purves, H. Brown, R. Haverty, A. Ryan, L.L. Griffin, J. McCormack, S. O’Reilly, P.W. Mallon, V. Gautier, J.P. Cassidy, A. Fabre, M.J. Carr, G. Gonzalez, S. Ciuti, N.F. Fletcher);; St Vincent's University Hospital, Dublin (P.W. Mallon, A. Fabre);; Hokkaido University, Sapporo, Japan (M.J. Carr, G. Gonzalez)

**Keywords:** SARS-CoV-2, COVID-19, coronavirus, respiratory infections, severe acute respiratory syndrome coronavirus 2, SARS, coronavirus disease, wildlife reservoir, deer, cervid, zoonoses, viruses, Dublin, Ireland

## Abstract

SARS-CoV-2 can infect wildlife, and SARS-CoV-2 variants of concern might expand into novel animal reservoirs, potentially by reverse zoonosis. White-tailed deer and mule deer of North America are the only deer species in which SARS-CoV-2 has been documented, raising the question of whether other reservoir species exist. We report cases of SARS-CoV-2 seropositivity in a fallow deer population located in Dublin, Ireland. Sampled deer were seronegative in 2020 when the Alpha variant was circulating in humans, 1 deer was seropositive for the Delta variant in 2021, and 12/21 (57%) sampled deer were seropositive for the Omicron variant in 2022, suggesting host tropism expansion as new variants emerged in humans. Omicron BA.1 was capable of infecting fallow deer lung type-2 pneumocytes and type-1–like pneumocytes or endothelial cells ex vivo. Ongoing surveillance to identify novel SARS-CoV-2 reservoirs is needed to prevent public health risks during human–animal interactions in periurban settings.

SARS-CoV-2, a member of the family Coronaviridae, is a positive-sense, single-stranded RNA (ssRNA) virus that has an ≈30-kb genome (*1*). During the COVID-19 pandemic, SARS-CoV-2 has caused ≈7 million deaths (*2*) and infected multiple mammalian species (*3*). Concerns have arisen regarding reverse zoonosis, during which the virus spills back from humans to animals, potentially leading to emerging new variants (*3*). High SARS-CoV-2 seroprevalence rates have been reported in white-tailed deer (*Odocoileus virginianus*) in the United States and Canada (*4*–*7*). Infected deer shed virus and transmit it to other deer; several lineages have been found to be similar to human SARS-CoV-2 genomes, raising the possibility of reverse zoonosis (*6*,*7*). This finding highlights the need to monitor wildlife, including deer, to understand whether those animals might serve as reservoirs for SARS-CoV-2 and pose a risk for transmission to other species (*8*).

Limited data exist on deer susceptibility to SARS-CoV-2 infection in Europe. As of 2021, surveillance reports from the United Kingdom, Austria, and Germany have not found evidence of SARS-CoV-2 exposure in fallow deer (*Dama dama*), red deer (*Cervus elaphus*), roe deer (*Capreolus capreolus*), or sika deer (*C. nippon*) (*9*,*10*). Whether infections are rare or nonexistent in those deer species remains uncertain. Although interactions between wild deer and humans might be low, angiotensin converting enzyme 2 (ACE2), a receptor for SARS-CoV-2, is expressed in bronchiolar epithelium of several deer species (*11*), suggesting that those species might be susceptible to SARS-CoV-2 infection (*12*). We conducted a SARS-CoV-2 surveillance study on a wild, free-ranging population of fallow deer in Europe’s largest urban park in Dublin, Ireland, during 2020–2022. We used quantitative reverse transcription PCR (qRT-PCR) and neutralization assays to detect SARS-CoV-2 virus in deer tissue and serum samples; in addition, we assessed the ability of ancestral and Omicron variants to infect fallow deer lung tissue and tracheal explants.

## Methods

### Study Area and Population

The study population of fallow deer is located in Phoenix Park, a 707-hectare urban park in Dublin (data from the Ireland Office of Public Works, https://www.gov.ie/en/organisation/office-of-public-works); a resident population of ≈600 free-ranging fallow deer exists in the park (*13*). The park receives up to 10 million visitors per year, and, since 2013, visitors have repeatedly been documented hand-feeding the deer population (*13*). The number of park visitors observed within 250 meters of deer herds during weekend observations was always >10 persons per deer group during 2019–2022: mean 19.7 (SD 14.7) persons in summer 2019, mean 21.9 (SD 22.8) in summer 2020, mean 15.7 (SD 18.0) in summer 2021, and mean 11.67 (SD 9.24) in summer 2022 (*13*). Phoenix Park is a critical site for biodiversity, supporting 50% of the wild mammal species found in Ireland and ≈40% of bird species (data from the Ireland Office of Public Works); mammals include badgers (*Meles meles*) and red foxes (*Vulpes vulpes*). Park visitors can bring their dogs into the park grounds, in most cases leashed, although unleashed dogs are permitted except during the deer fawning season.

The behavior of the deer and their interactions with humans have been extensively characterized, and >80% of the population has been identified by using ear tags (*14*). Areas of the park accessible to the public are used by 86% of the deer; of those, 24% have a high contact rate with humans that includes taking food (consistent beggars), 68% display intermediate deer–human contact rates (occasional beggars), and 8% systematically avoid any interactions with humans despite living in areas open to the public (rare beggars). The remaining 14% (avoiders) avoid areas of the park accessible to humans (*13*,*15*). The interaction categories are defined on the basis of begging rank, which is a scale of most to least likely to beg according to previously generated models of begging behavior (Appendix 1) (*13*). Deer are culled annually by professional deer stalkers over the winter period; the deer stalkers aim to maintain a population that mimics a natural structure.

### Sample Collection and Storage

Culling of fallow deer occurred on November 2 and 25, 2021, and February 16, 2022; in addition, we collected archived serum samples from November 2020 (Appendix 2 Table 1). We collected retropharyngeal lymph nodes, palatine tonsil, nasopharyngeal mucosa and cecal content within 1 hour after death and blood samples immediately after death. We chose the sampling strategy according to the highest viral loads reported from experimental infection of white-tailed deer (*7*). We did not collect nasopharyngeal swab samples from the deer; we elected to directly sample nasopharyngeal mucosal tissues postmortem. We stored tissue samples and cecal content at −80°C before RNA extraction and serum samples at −20°C.

### SARS-CoV-2 Surrogate Virus Neutralization Test

We performed SARS-CoV-2 surrogate virus neutralization tests (sVNTs) on deer serum samples by using the Genscript cPass SARS-CoV-2 sVNT Kit (Genescript, https://www.genscript.com) according to the manufacturer’s instructions (Appendix 1). We screened serum samples in duplicate within each assay and performed 2 independent assays, expressing results as percent neutralization.

### Nucleic Acid Extraction and SARS-CoV-2 qRT-PCR

We isolated total RNA from tissues by homogenizing in TRIzol, extracting the aqueous layer, and then using the RNeasy Mini Kit (QIAGEN, https://www.qiagen.com) according to the manufacturer’s instructions. We spiked the tissue RNA samples with 1 µg MS2 bacteriophage RNA as an extraction control. We isolated RNA from cecal content samples as previously described (Appendix 1) (*16*) and spiked those samples with 100 µL of murine hepatitis virus (4.22 × 10^7^ 50% tissue culture infectious dose [TCID_50_]/mL) as extraction controls. We performed qRT-PCR on an ABI 7500 Real-Time PCR System (Thermo Fisher Scientific, https://www.thermofisher.com) by using Taqman Fast Virus 1-Step Master Mix (Thermo Fisher Scientific) and specific oligonucleotide primer and probe sequences and thermocycling conditions (Appendix 1 Table). We used EURM-019, a synthetic SARS-CoV-2 ssRNA obtained from the European Commission Joint Research Centre (https://joint-research-centre.ec.europa.eu), as a standard for SARS-CoV-2 envelope (E) gene quantification. We analyzed all samples and controls in triplicate.

### Cell Culture, SARS-CoV-2 Pseudovirus, and Infectious Virus Neutralization Test

We propagated untransfected Vero E6 cells (American Type Culture Collection, https://www.atcc.org) and Vero E6 cells transiently expressing an untagged transmembrane protease, serine 2 (TMPRSS2) cDNA expression vector (Sino Biological, https://www.sinobiological.com) as previously described (*17*). We generated SARS-CoV-2 pseudoviruses bearing spike proteins from Alpha, Delta, Omicron BA.1, and Omicron BA.2 variants (InvivoGen, https://www.invivogen.com) as previously described (*18*). In brief, we co-transfected 293T cells with plasmids encoding an HIV-1 provirus expressing luciferase (pNL4-3-Luc-R-E; National Institute for Biologic Standards and Control, https://nibsc.org) and either vesicular stomatitis virus glycoprotein, SARS-CoV-2 spike protein, or a no E control. We harvested supernatants 48 and 72 hours after transfection, filtered them through a 0.45 µm filter, and stored them at −80°C.

We propagated SARS-CoV-2 strain 2019-nCoV/Italy-INMI1 (European Virus Archive Global, https://www.european-virus-archive.com; GenBank accession no. MT077125.1) in Vero E6 cells as previously described (*17*). We performed TCID_50_ assays of Vero E6 cells in quadruplicate and determined infectious titers as previously described (*19*). We isolated SARS-CoV-2 Omicron BA.1 (GenBank accession no. ON350968, passage 2) from a SARS-CoV-2–positive nasopharyngeal swab sample obtained during the All Ireland Infectious Disease Cohort Study (P.W.G. Mallon et al., unpub. data, https://doi.org/10.1101/2021.02.09.21251402) and amplified the virus on Vero E6/TMPRSS2–expressing cells obtained from the Centre For AIDS Reagents at the National Institute for Biologic Standards and Control (*20*).

We incubated individual pseudoviruses or infectious viruses with deer serum samples at a 1:1 ratio for 1 hour at 37°C and then titrated the viruses on Vero E6/TMPRSS2 cells. For pseudovirus infections, we lysed the cells after 48 hours by using Passive Lysis Buffer (Promega, https://www.promega.com) and quantified luciferase activity by using a TriStar^2^ LB 942 Multimode Reader (Berthold Technologies, https://www.berthold.com). We expressed infectivity as relative light units, minus the no-E control signal, relative to the virus only control. For infectious virus assays, we scored the cytopathic effect 48 hours after infection and calculated TCID_50_ (*19*). To differentiate between cytopathic effect and cytotoxicity following incubation with serum samples, we titrated each serum sample alone on Vero E6/TMPRSS2 cells and scored cytotoxicity 48 hours after inoculation; we only scored dilutions that had no visible cytotoxic effect (i.e., rounded or detached cells) in TCID_50_ assays.

### SARS-CoV-2 Superlineages Circulating during Sampling Months

We performed SARS-CoV-2 whole-genome sequencing of human clinical samples collected in Ireland as described previously (*21*), covering 1-month periods concurrent with the deer culling dates; human sequences were downloaded from GISAID (https://www.gisaid.org). We assigned lineages by using the Pangolin application version 4.3 and Pangolin data version 1.20 (https://github.com/cov-lineages/pangolin) and assigned superlineages by using the most recent common ancestors of SARS-CoV-2 variants B.1, B.1.177, B.1.1.7 (Alpha), P.2 (Zeta), B.1.617.2 (Delta), B.1.1.529.1 (Omicron BA.1), B.1.1.529.2 (Omicron BA.2), and B.1.1.529.3 (Omicron BA.3). For graphical representations, we sampled 108 genomes proportionally to their superlineage frequencies from all 3 sampling periods.

We aligned multiple sequences of the SARS-CoV-2 genome sequences from humans with a GenBank reference sequence (accession no. MN908947) by using the MAFFT program and FFT-NS-I algorithm (*22*). We inferred a phylogenetic tree by aligning sequences (n = 109) by using the program RAxML (https://antonellilab.github.io/raxmlGUI) and a general time-reversible substitution model and estimated branch support by using 100 bootstrap replicates.

### SARS-CoV-2 Infection of Tracheal Explants and Precision Cut Lung Slices

We generated precision cut lung slices (PCLSs) and tracheal explants from 2 SARS-CoV-2–seronegative fallow deer and inoculated the tissues with either SARS-CoV-2 Italy-INMI-1 (ancestral virus) or Omicron BA.1 strains (Appendix 1). For tracheal infections, we added 100 μL virus (Italy-INMI-1 titer, 3.9 × 10^5^ TCID_50_/mL; Omicron BA.1 titer, 1.3 × 10^3^ TCID_50_/mL) to the epithelial surface of tracheal tissue; for PCLS infections, we added 500 μL virus and 500 μL culture medium to each well containing lung tissue. We infected tissue with each virus variant in triplicate. We removed virus 24 hours after inoculation, washed the tissue in phosphate-buffered saline, replenished the culture medium, and cultured the tissue for another 48 hours. Then, we fixed tissue with 10% neutral-buffered formalin.

### Immunohistochemistry to Detect SARS-CoV-2 Antigen

To evaluate SARS-CoV-2 expression profiles, we immunohistochemically stained formalin-fixed, paraffin-embedded tissue sections (Appendix 1) by using mouse monoclonal IgG1 antibody against SARS-CoV-2 spike protein (GeneTex, https://www.genetex.com) and the EnVision Flex kit (Agilent Technologies Inc., https://www.agilent.com). We scanned the slides by using the Aperio AT2 digital slide scanner and reviewed images by using Aperio ImageScope 12.4 software (both Leica Biosystems, https://www.leicabiosystems.com).

### Statistical Analyses

We expressed in vitro results as means +SD, except as indicated, and analyzed data by using 1-way analysis of variance in Prism 9.0 (GraphPad, https://www.graphpad.com). We estimated individual begging behavior as previously described (Appendix 1) (*13*).

## Results

### SARS-CoV-2–Seropositive Fallow Deer

**Figure 1:**
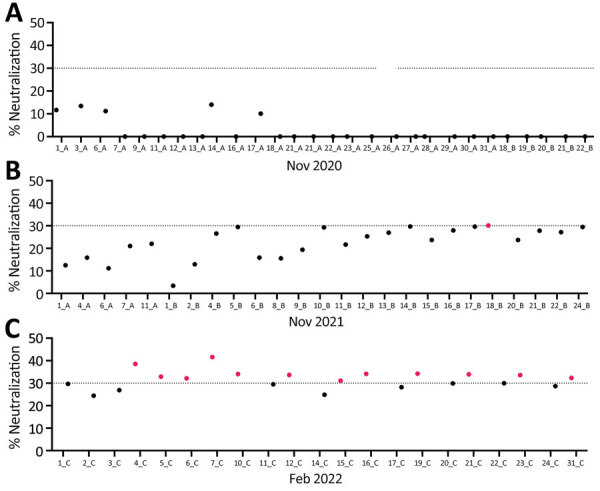
SARS-CoV-2 neutralizing antibodies found in serum samples from fallow deer in an urban deer population located in Dublin, Ireland, 2020–2022. A) Serum samples collected in November 2020 (n = 28); B) samples from November 2021 (n = 25); C) samples from February 2022 (n = 21). Serum samples were collected from wild fallow deer and screened in duplicate for SARS-CoV-2 neutralizing antibodies by using the Genscript cPass SARS-CoV-2 surrogate virus neutralization test (Genescript, https://www.genscript.com). Deer identification numbers are shown on the x axes for each year. Dotted lines indicate a cutoff of 30% neutralization. Red dots indicate serum samples that had >30% neutralization and were considered seropositive for SARS-CoV-2. Data are presented as mean percent neutralization calculated from duplicate wells from 2 independent assays.

Using an sVNT specific for SARS-CoV-2, we screened serum samples from fallow deer culled in November 2020 (n = 28), November 2021 (n = 25), and February 2022 (n = 21) for SARS-CoV-2 neutralizing antibodies. All deer culled in November 2020 had sVNT neutralization cutoff values of <30% and were considered seronegative. All deer, except 1 from the November 2021 cull, were also seronegative. Deer no. 18_B_2021, culled on November 25, 2021, had an sVNT neutralization value of 30% and was considered seropositive. Twelve of 21 (57%) deer culled in February 2022 had neutralization values >30% and were considered seropositive for SARS-CoV-2 (Table; Figure 1). SARS-CoV-2 seropositive animals ranged from <1 (fawn) to 8 years of age and were from various subherds within the park (Table). Of the 13 seropositive deer, 10 (77%) were male and 3 (23%) female (Table).

**Table:** Profiles and SARS-CoV-2 PCR status of seropositive fallow deer in study of SARS-CoV-2 seropositivity in urban population of wild fallow deer, Dublin, Ireland, 2020–2022*

Animal code	Sample date	Age, y/sex	Neutralization, %†	Tissue PCR	Cecal content PCR	Begging category‡
18_B_2021	2021 Nov 25	4/M	30	ND	ND	Unranked
4_C_2022	2022 Feb 16	1/M	39	ND	ND	Occasional
5_C_2022	2022 Feb 16	6/F	33	ND	ND	Occasional
6_C_2022	2022 Feb 16	<1/M	32	ND	ND	Unranked
7_C_2022	2022 Feb 16	1/M	42	ND	ND	Occasional
10_C_2022	2022 Feb 16	8/F	34	ND	ND	Occasional
12_C_2022	2022 Feb 16	2/M	34	ND	ND	Unranked
15_C_2022	2022 Feb 16	3/F	31	ND	ND	Occasional
16_C_2022	2022 Feb 16	3/M	34	ND	ND	Consistent
19_C_2022	2022 Feb 16	7/M	34	ND	ND	Unranked
21_C_2022	2022 Feb 16	5/M	34	ND	ND	Consistent
23_C_2022	2022 Feb 16	5/M	34	ND	ND	Unranked
31_C_2022	2022 Feb 16	1/M	32	ND	ND	Unranked

*SARS-CoV-2 seropositive fallow deer sampled in February 2022 (n = 21) were PCR negative for the SARS-CoV-2 envelope gene in all samples tested (retropharyngeal lymph nodes, nasopharyngeal mucosa, palatine tonsil, and cecal content). ND, not detected. †SARS-CoV-2 surrogate virus neutralization test was used to determine % neutralizing antibodies in deer serum samples. ‡Begging ranks were occasional or consistent beggars or unranked. Unranked indicates the deer were untagged.

### SARS-CoV-2 Pseudovirus and Infectious Virus Neutralization

**Figure 2:**
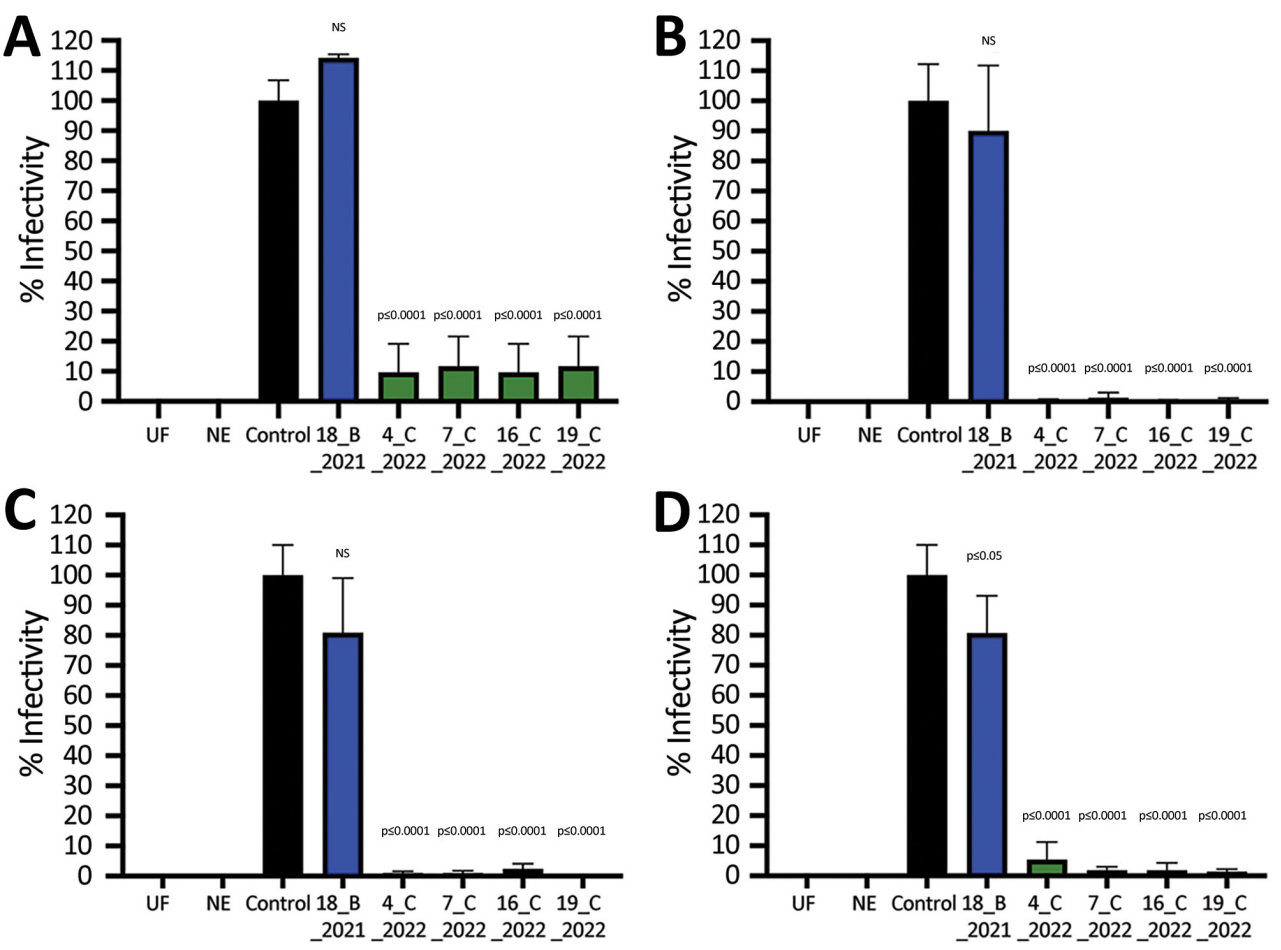
Infectivity of SARS-CoV-2 pseudoviruses after incubation with SARS-CoV-2–positive serum samples from wild fallow deer, Dublin, Ireland, 2020–2022. Spike proteins were from Alpha (A), Delta (B), Omicron BA.1 (C), and Omicron BA.2 (D) variants of concern. SARS-CoV-2 pseudoviruses bearing spike proteins from different variants of concern were incubated with 5 deer serum samples at a 1:1 ratio in triplicate and then used to infect Vero E6/TMPRSS2 cells. Identification numbers of deer are indicated. Controls were virus incubated in triplicate at a 1:1 ratio with culture medium. Relative light units from a luciferase reporter were used to calculate percentage infectivity relative to the untreated control virus. Data are from 2 independent experiments with 3 biologic replicates per experiment. Error bars indicate SDs. NE, no envelope naked pseudovirus control; NS, not significant; UF, uninfected cells.

**Figure 3:**
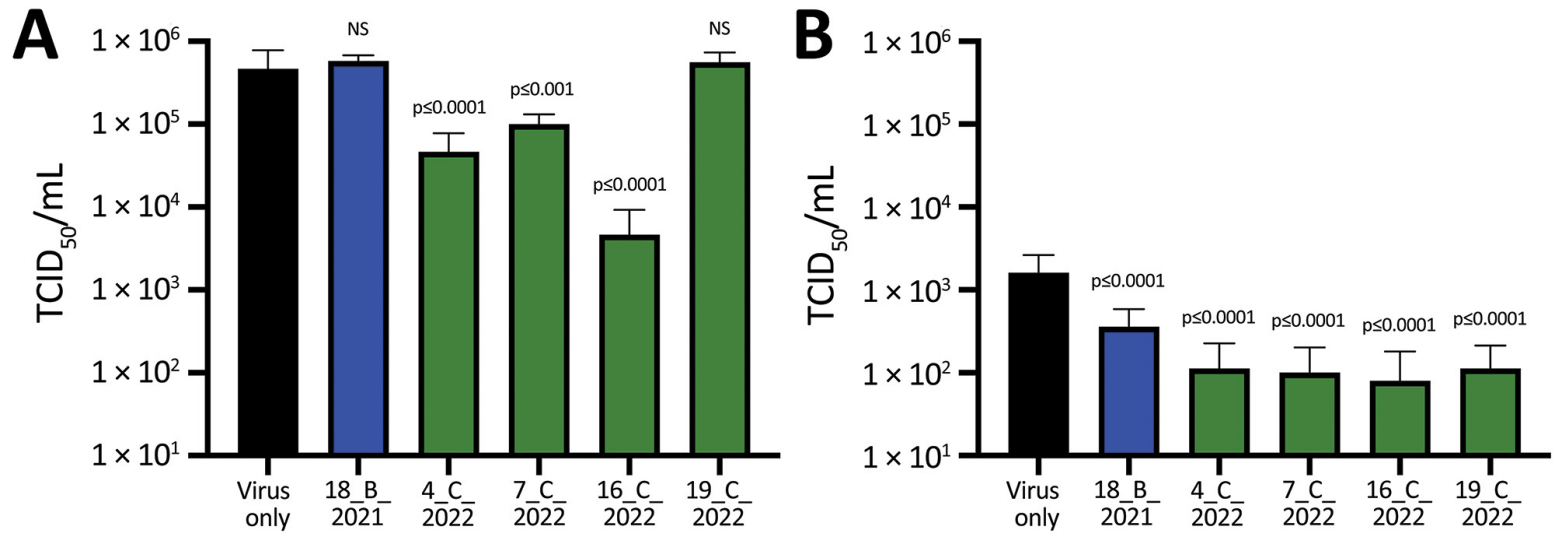
Infectivity of SARS-CoV-2 infectious viruses after incubation with SARS-CoV-2–positive serum samples from wild fallow deer, Dublin, Ireland, 2020–2022. Deer serum samples were incubated with infectious SARS-CoV-2 ancestral strain Italy_INMI1 (A) or Omicron BA.1 (B) and then used to infect Vero E6/TMPRSS2 cells. Identification numbers of deer are indicated. Cytopathic effect was calculated as TCID_50_, as previously described (*19*). Data are from 2 independent experiments with 8 biologic replicates per experiment. p values were calculated by using 1-way analysis of variance (Appendix 2 Tables 2, 3). Error bars indicate SDs. NS, not significant; TCID_50_, 50% tissue culture infectious dose.

To confirm the ability of sVNT-positive deer serum samples to neutralize SARS-CoV-2 pseudovirus and infectious virus, we selected 4 serum samples with the highest sVNT neutralization titers from the February 2022 cull together with a seropositive sample from the November 2021 cull (deer no. 18_B_2021). All SARS-CoV-2–seropositive serum samples from 2022 neutralized pseudoviruses bearing Alpha, Delta, Omicron BA.1, and BA.2 spike proteins, whereas the sample from deer no. 18_B_2021 only significantly inhibited pseudovirus containing the BA.2 spike protein (Figure 2). None of the 5 serum samples neutralized vesicular stomatitis virus glycoprotein from an unrelated pseudovirus construct. Using infectious ancestral SARS-CoV-2 (Italy-INMI1) and Omicron BA.1 viruses, we observed significant inhibition of the ancestral virus by 3 of 5 serum samples and Omicron BA.1 by all 5 serum samples tested (Figure 3; Appendix 2 Tables 2, 3).

### Fallow Deer qRT-PCR Negative for SARS-CoV-2

All animals were PCR negative for the SARS-CoV-2 E gene in tissues collected during November 2021 and February 2022, regardless of serum status (Table; Appendix 1 Table). SARS-CoV-2 ssRNA controls were amplified in all assays, indicating the qRT-PCR amplified the SARS-CoV-2 E gene correctly.

### Begging Behavior of SARS-CoV-2 Seropositive Deer

**Figure 4:**
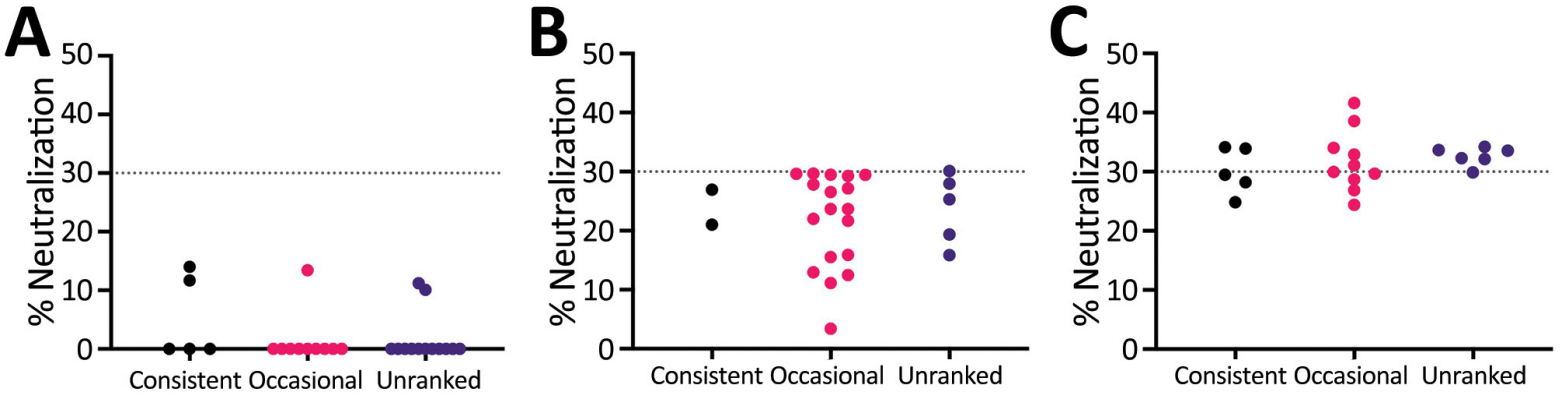
Begging behavior of deer sampled to detect SARS-CoV-2 neutralizing antibodies in study of SARS-CoV-2 seropositivity in urban population of wild fallow deer, Dublin, Ireland, 2020–2022. A) November 2020; B) November 2021; C) February 2022. Dotted lines indicate a cutoff of 30% neutralization of SARS-CoV-2 by serum antibodies; >30% neutralization was considered SARS-CoV-2 seropositive. Red dots indicate occasional beggars; most deer were either consistent or occasional beggars.

**Figure 5:**
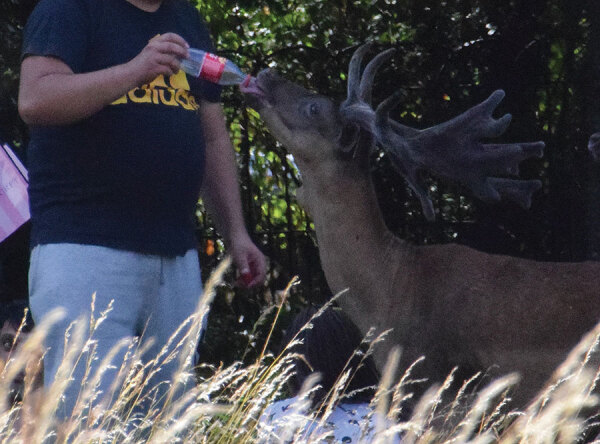
Example of fallow deer–human interaction in study of SARS-CoV-2 seropositivity in urban population of wild fallow deer, Dublin, Ireland, 2020–2022. Photograph by Bawan Amin, University College Dublin, July 2018.

**Figure 6:**
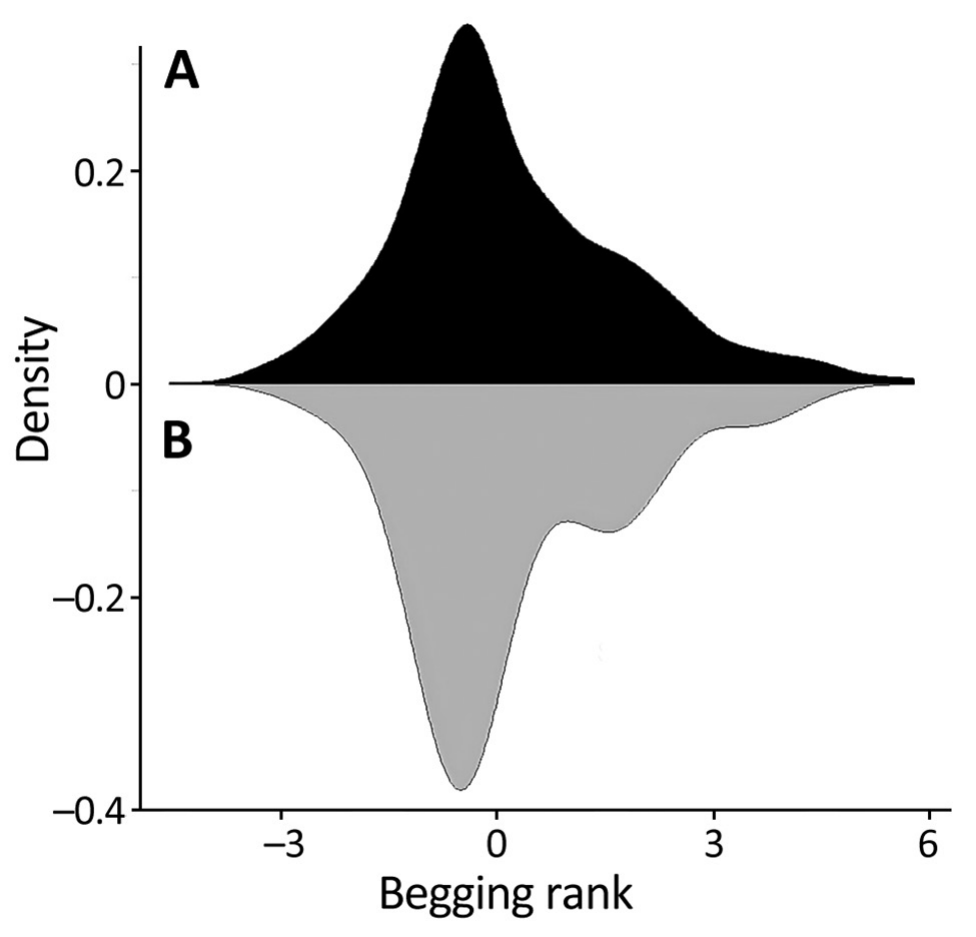
Deer begging rank distributions in study of SARS-CoV-2 seropositivity in urban population of wild fallow deer, Dublin, Ireland, 2020–2022. Mirror density plot was generated to compare begging rank distributions (Appendix 1) for the whole deer population (black shading) and sampled deer (gray shading).

**Figure 7:**
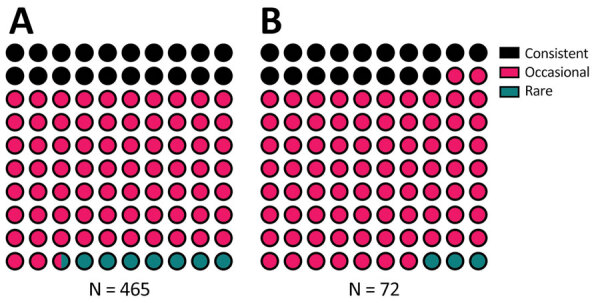
Begging category proportions in study of SARS-CoV-2 seropositivity in urban population of wild fallow deer, Dublin, Ireland, 2020–2022. A) Total fallow deer population; B) fallow deer sampled for SARS-CoV-2 serum antibodies.

**Figure 8:**
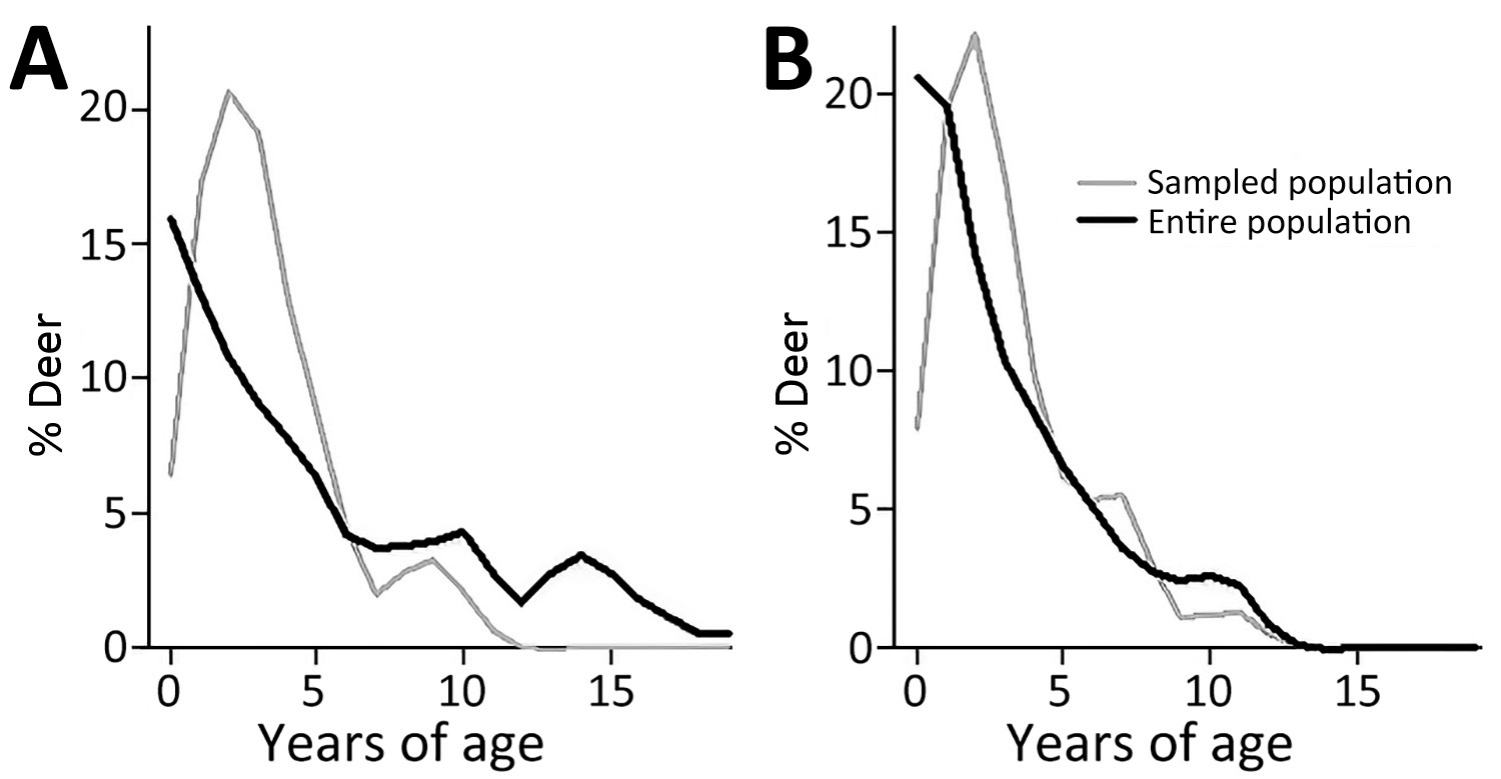
Deer age and sex structure in study of SARS-CoV-2 seropositivity in urban population of wild fallow deer, Dublin, Ireland, 2020–2022. A) Female deer; B) male deer.

Most sampled deer were consistent or occasional beggars (Figure 4) (*13*). Of the seropositive animals sampled during February 2022, 7/12 (58%) were consistent or occasional beggars; the remaining 6 seropositive deer from both 2021 and 2022 were unranked, indicating they were either not tagged or tagged without an assigned rank, as in the case of a fawn (deer no. 6_C_2022). However, the fawn’s mother is a documented consistent beggar; therefore, it is possible that this fawn came in contact with humans while following its mother. Beggars took various foodstuffs from humans (Figure 5) (*13*). The sampled deer represented the entire fallow deer population in terms of begging rank (Figure 6), begging category (Figure 7), and age and sex classes (Figure 8). We attempted to link begging rank to serum status by using a regression model that had the year of study and begging rank as predictors and serum status as the response variable. Although we observed a positive relationship between the variables, not enough power was achieved for a significant p value, because many seropositive animals were untagged.

### SARS-CoV-2 Superlineages Circulating in Human Population 

**Figure 9:**
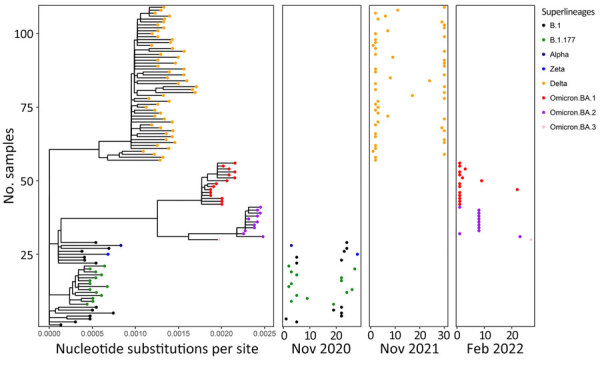
Phylogenetic analysis of SARS-CoV-2 superlineages circulating in humans during deer sampling months in study of SARS-CoV-2 seropositivity in wild fallow deer, Dublin, Ireland, 2020–2022. We analyzed SARS-CoV-2 whole-genome sequences from human clinical samples collected in Ireland covering months corresponding to the deer culling dates (November 2020, November 2021, and February 2022). Branch lengths in the phylogenetic tree (left panel) show the number of base substitutions per site. Colors indicate different SARS-CoV-2 variants. Pangolin lineages are shown with corresponding major circulating variants for each cull month. Location of dots shown for each cull month (right 3 panels) corresponds to the sampling date in each month (horizontal axis) and the phylogenetic position within the tree panel (vertical axis).

The SARS-CoV-2 genome sequences (n = 5,012) from human clinical samples obtained during November 2020 (n = 224), November 2021 (n = 2,883), and February 2022 (n = 1,905) enabled the examination of variants circulating in the human population during the fallow deer cull periods (Figure 9). The genome sequences from clinical samples showed a clear demarcation between different variant waves and nonoverlapping phylogenetic relationships among the lineages identified during the 3 deer sampling periods. During the first sampling period (November 2020), B.1 and B.1.177 were the main lineages circulating among the population in Ireland; Alpha and Zeta variants were detected during this month, and the Alpha variant was predominant during the following months (P.W.G. Mallon, et al., unpub. data). During November 2021, the Delta variant was the main circulating variant for the entire month, whereas in February 2022, Omicron variants BA.1 and BA.2 were the main variants detected; Omicron BA.3 was detected at the end of February 2022.

### Fallow Deer Trachea and Lung Tissues Infected by SARS-CoV-2

**Figure 10:**
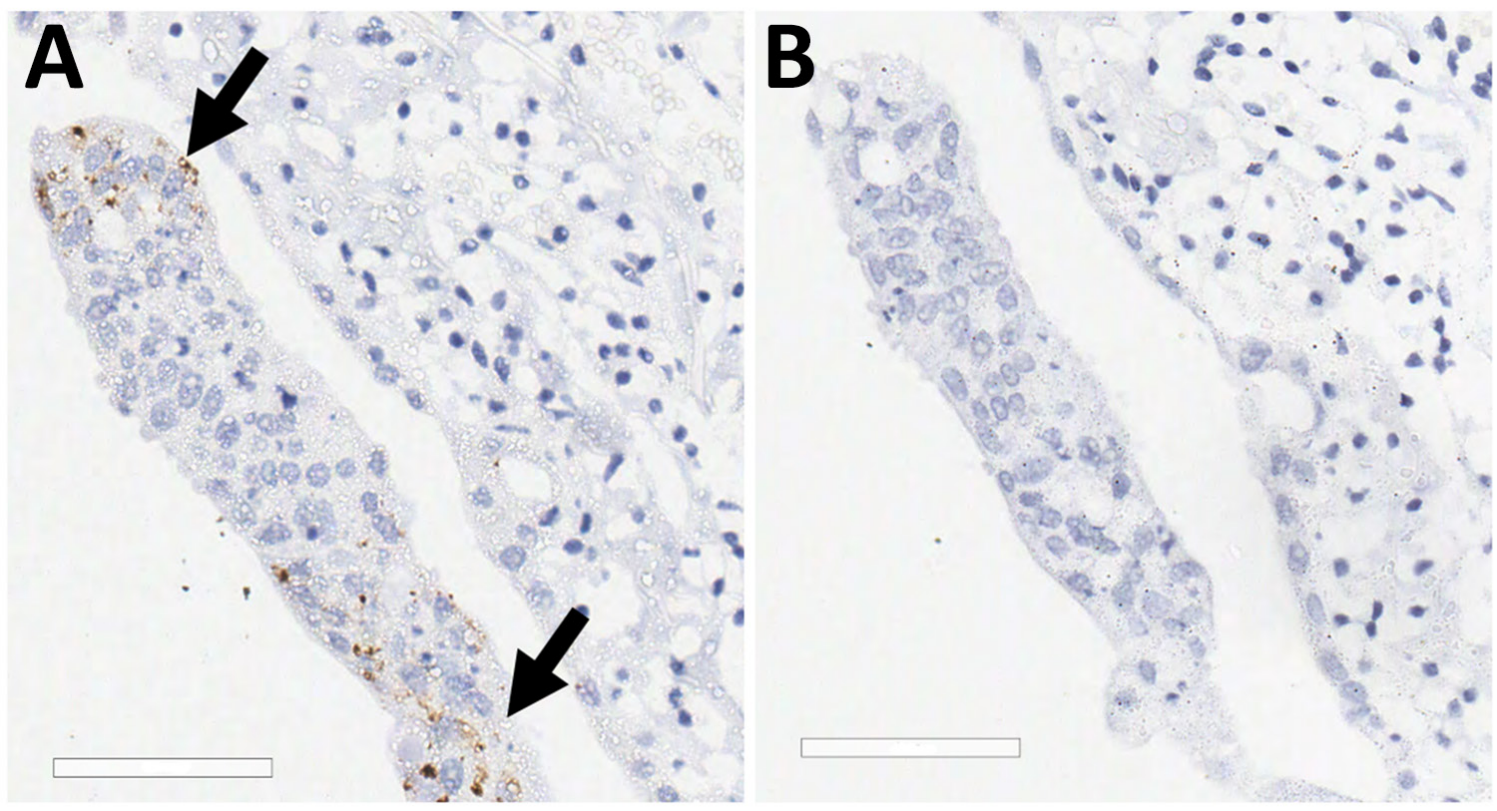
SARS-CoV-2 infection of tracheal explant in study of SARS-CoV-2 seropositivity in urban population of wild fallow deer, Dublin, Ireland, 2020–2022. Tracheal explants from 2 SARS-CoV-2–seronegative deer were inoculated with SARS-CoV-2 Italy-INMI1 and stained by using immunohistochemistry. Control sections were stained with IgG only or mock infected. A) Arrows indicate SARS-CoV-2 Italy-INMI1 antigen immunoreactivity in tracheal epithelium; B) no immunoreactivity was observed after staining with the IgG control. Scale bars indicate 60 μm.

**Figure 11:**
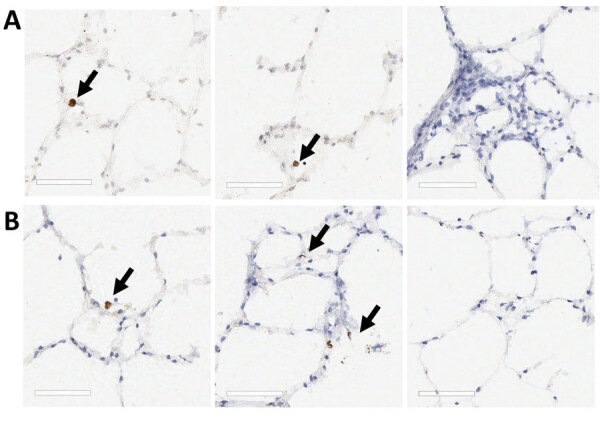
SARS-CoV-2 Omicron BA.1 infection of ex vivo lung tissue in study of SARS-CoV-2 seropositivity in urban population of wild fallow deer, Dublin, Ireland, 2020–2022. Precision cut lung slices were collected from 2 SARS-CoV-2–seronegative deer and inoculated with SARS-CoV-2 Omicron BA.1; sections were stained by using immunohistochemistry. Control sections were stained with IgG only or mock infected. A) Deer 1; B) deer 2. Arrows in first and middle panels indicate Omicron BA.1 immunoreactivity in cells morphologically consistent with type 2 pneumocytes. Third panel indicates no immunoreactivity after staining with the IgG control. No immunoreactivity was observed in the mock-infected tissues for either animal. Scale bars indicate 60 μm.

Two board-certified pathologists (J.P.C. and A.F.) reviewed the tissue slides without having prior knowledge of sample treatment and assessed the presence or absence of SARS-CoV-2 staining and the likely cells showing immunoreactivity. Tracheal epithelium inoculated with Italy-INMI1 (ancestral SARS-CoV-2), but not Omicron, was antigen-positive in 2 of 3 experimental replicates from 1 deer (Figure 10). In contrast, in lung tissue, cells morphologically consistent with type 2 pneumocytes and elongated cells representing type 1 pneumocytes or endothelial cells were immunoreactive in all 3 replicates from animals inoculated with Omicron BA.1, but not Italy-INMI1 (Figure 11). No immunoreactivity was observed in tracheal or lung tissue stained with the IgG control or in mock-infected tissue (Figure 11). We attempted to confirm infection by using qRT-PCR for the SARS-CoV-2 E gene 24–72 hours postinfection but were unable to distinguish between residual input virus and de novo virus released from infected PCLSs.

## Discussion

The World Health Organization, World Organisation for Animal Health, and Food and Agriculture Organization of the United Nations have emphasized the need to monitor SARS-CoV-2 in wildlife because of the potential establishment of animal reservoirs and possible generation of novel variants (*23*). Minimizing transmission between humans and wildlife has also been emphasized, including educating the public about the risks of contact with wild animals (*24*).

White-tailed deer and mule deer are the only deer species reported to be susceptible to SARS-CoV-2 infection (*5*,*24*). White-tailed deer shed infectious virus, leading to deer-to-deer and deer-to-human transmission (*7*,*25*). Other mammal species capable of transmitting SARS-CoV-2 are mink, raccoons, dogs, cats, ferrets, hamsters, mice, Egyptian fruit bats, and deer mice (*3*). In contrast to white-tailed and mule deer, fallow deer (both *Dama dama* and *D. mesopotamica*) are a monophyletic clade within Old World deer (*26*). Human interactions with the fallow deer population in Phoenix Park have increased since 2013 because of higher social media visibility of the deer in the Park and the animals’ willingness to take food from humans (*13*). During the COVID-19 pandemic, human–deer interactions rose in 2020 because of increased park usage for recreation during lockdown, but interactions returned to prepandemic levels by summer 2021 (*27*). 

We identified 13 seropositive deer that showed 34% mean SARS-CoV-2 neutralization in the sVNT; the highest neutralization was 42%. Although this sVNT has not been specifically validated for deer species, the same sVNT has been used to report neutralization levels approaching 80%–90% for white-tailed deer (*4*), and the assay has been assessed in other species susceptible to SARS-CoV-2 infection, such as hamsters, mink, ferrets, and cats (*28*). In this study, SARS-CoV-2 neutralization levels using pseudoviruses were higher than those from the Genescript sVNT assays used for serum samples from February 2022 and the positive serum sample from November 2021 (deer 18_B_2021). The serum sample from deer 18_B_2021 significantly inhibited the BA.2 pseudovirus. All serum samples were capable of neutralizing infectious Omicron BA.1, but only 3 of 5 significantly neutralized infectious virus strain Italy_INMI1. 

Because the sVNT uses the SARS-CoV-2 spike protein from the ancestral virus, it is possible that more seropositive serum samples might have been detected if the sVNT had incorporated later mutated variants (*29*). This sVNT result, together with other studies, suggests the sVNT should be interpreted qualitatively rather than quantitatively (*28*). The sVNT specifically detects SARS-CoV-2 neutralizing antibodies and has no cross-reactivity with other human coronaviruses or respiratory viruses or Middle East respiratory syndrome coronavirus (*28*) except SARS-CoV, which is closely related antigenically to SARS-CoV-2, according to cPass SARS-CoV-2 sVNT Kit documentation (Genescript). During the fallow deer sampling period, no evidence of SARS-CoV circulation existed in Ireland. However, although respiratory disease was not observed in the study population, cross-reactivity with other animal coronaviruses in the sVNT, including bovine coronavirus, is possible but has not been evaluated. It is unclear whether bovine coronavirus is circulating in this population (*30*), but other respiratory viruses have a low seroprevalence in deer in Ireland (*31*).

SARS-CoV-2–seropositive deer in this study were mainly occasional beggars, which reflected the begging category distribution of the entire deer population (*13*). We only sampled 2 rare beggars or avoiders in this study, likely because of their contact avoidance with humans and, therefore, their lower likelihood of being culled, which agrees with previous studies that shyer animals evade observation and trapping (*13*,*32*). Overall, 77% of seropositive deer were male, consistent with the hypothesis of anthroponosis, because male deer are more likely to beg for food (*13*).

During the 3 sampling periods, diverse SARS-CoV-2 variants circulated in the human population (*22*; A.M. Rice et al., unpub. data, https://doi.org/10.1101/2023.05.11.23289783). Because of the high frequency of interactions with humans, it is likely that the fallow deer population was exposed to SARS-CoV-2 through human contact. Although the February 2022 deer sampling period coincided with circulation of the Omicron variant within the human population, it is not possible to definitively state to which subvariant the seropositive deer in this study were exposed or whether SARS-CoV-2 transmission occurred between deer. SARS-CoV-2 surveillance studies in Europe have not sampled animals after 2021 or studied deer that had defined interactions with humans (*9*,*10*). Our study highlights the need for ongoing SARS-CoV-2 surveillance in animals, particularly as novel variants of concern are still emerging (*23*).

We showed that fallow deer PCLS tissue supported SARS-CoV-2 infection with Omicron BA.1 but not the ancestral virus. Omicron-positive cells were observed in PCLS tissue 72 hours after infection, and those cells expressed ACE2 (*11*). Whether other receptors, such as TMPRSS2, are necessary for SARS-CoV-2 infection in fallow deer remains unknown. Infected cells were observed in tracheal epithelium from 1 of 2 animals inoculated with ancestral SARS-CoV-2 but not Omicron. The reasons for differences in tissue distribution are unclear, and further studies will be needed to determine tissue distribution at different timepoints after infection (*33*,*34*).

None of the deer sampled in this study were PCR positive for SARS-CoV-2, and no clinical signs were observed. However, because of the short duration of viral replication compared with the extended persistence of antibodies in white-tailed deer (>13 months), we were more likely to detect seropositive animals than those shedding virus (*35*). Although an annotated fallow deer genome is not available, ACE2 residues binding SARS-CoV-2 spike protein are conserved across cervids and have high homology to human ACE2 (*36*). Moreover, deer species for which ACE2 sequences are not available, including *D. dama*, are also likely to have conserved key ACE2 residues (*36*). Our findings suggests that the SARS-CoV-2 Omicron variant might infect fallow deer lung tissue in contrast to ancestral SARS-CoV-2, highlighting the importance of ongoing deer surveillance.

In conclusion, we report SARS-CoV-2 seropositivity in fallow deer in Ireland. In February 2022, Omicron was the dominant variant in humans, and 57% of fallow deer were also seropositive for SARS-CoV-2. Ongoing surveillance to identify novel reservoirs of SARS-CoV-2 and other zoonotic pathogens is needed to prevent ecologic public health risks for human–animal interactions in periurban settings.

Appendix 1 Additional information for SARS-CoV-2 seropositivity in urban population of wild fallow deer, Dublin, Ireland, 2020–2022.

Appendix 2 Detailed information for sampled deer and statistical analysis of infectivity of infectious viruses after neutralization by SARS-CoV-2–positive serum samples.
