# Supplementary material for: SARS-CoV-2 Seropositivity in Urban Population of Wild Fallow Deer, Dublin, Ireland, 2020–2022
Source: Emerg Infect Dis. 2024 Aug;30(8):1609–20. doi: 10.3201/eid3008.231056 (PMC11286063; doi:10.3201/eid3008.231056)
Supplement: Appendix 1 — Additional information for SARS-CoV-2 seropositivity in urban population of wild fallow deer, Dublin, Ireland, 2020–2022. [file 23-1056-Techapp-s1.pdf]

Article DOI: https://doi.org/10.3201/eid3008.231056

EID cannot ensure accessibility for supplementary materials supplied by authors. Readers who have difficulty accessing supplementary content should contact the authors for assistance.

SARS-CoV-2 Seropositivity in Urban Population of Wild Fallow Deer, Dublin, Ireland, 2020–2022

Appendix 1

Additional Methods

Begging ranks and categories

Step 1, Modeling Begging Ranks

Data analyses were performed in R version 4.0.5 (The R Project for Statistical Computing, https://www.r-project.org). Feeding data was collected as previously described (*1*), and all deer observed for <1,080 minutes (i.e., 3 observation sessions) were dropped from the analysis to remove potential noise from underrepresented animals. Our final dataset was composed of 19,451 rows, encompassing data from feeding collections performed from the start of June to end of July (Friday–Sunday) during 2019–2021. Each row in our dataset corresponded to an observation for an identified deer, the code for the herd to which they belonged, whether or not they engaged in an interaction (binomials: 1 = a line for each time the deer engaged in a separate feeding interaction over the course of that herd observation or 0 = the deer were documented as being present in the herd but never approached any available feeding interactions), information on the interaction (i.e., how many persons were involved), and all relevant spatial and temporal information (*1*).

We fit a generalized linear mixed-effects model with binomial distribution of errors following a priori structure by using *glmer* function in the *lme4* R package (*2*); begging was a response variable and deer and herd identification (ID) numbers (observation number) were crossed random intercepts. Our model a priori structure was built by using predictors of interest that we selected before the beginning of data collection. We included deer age, sex, herd size, the

total number of persons that attempted to interact with the herd during the observation session, the day of the week (categorical: Friday, Saturday, Sunday), the time of day, the amount of time the observer spent monitoring the herd (i.e., sampling effort), the month of observation (categorical: June, July) and year of study (categorical: 2019, 2020, 2021) as explanatory variables in our model. All numerical predictors were scaled to improve model convergence and were included as both single and quadratic terms in the model to factor in nonlinear patterns.

We also included 2-way interactions in the model as follows: sex and age, sex of the deer and total number of persons attempting to interact with the herd, and sex and herd size, all of which were included in both their linear and quadratic forms leading to a total of 6 interactions. Variable selection rationale and a priori expectations are detailed as previously described (*1*). All predictors included in the model structure were not collinear (Pearson's correlation coefficient $r_p<0.7$) (*3*). We then extracted the random intercepts estimated by the generalized linear mixed-effects model for each individual ID, also known as conditional modes or best linear unbiased predictors (BLUPs) (*4*), which were highly repeatable across animals (*1*,*5*). This model constituted a ranking system, which ranged from lowest to highest deer begging rates after taking all model predictors into account.

Step 2, Extracting Begging Categories

We extracted the begging ranks (i.e., random intercept or BLUP value) and calculated 95% CIs for each random intercept value for each deer ID and then summarized them into behavioral categories. The behavioral categories were identified depending on how the random effects and related CIs were distributed around the median random effect (zero, i.e., the median begging behavior of the population). Ultimately, begging behavior exists on a continuum, but we subdivided those behaviors into 3 categories for clarification: random effect 95% CI >0 (consistent beggars), 95% CI overlapped 0 (occasional beggars), or 95% CI <0 (rare beggars). This approach considers and categorizes the BLUPs (random intercept) and connects them with the associated error (CIs) (*6,7*). Those begging categories were then associated with the ID of each culled deer.

SARS-CoV-2 Surrogate Virus Neutralization Test

We performed SARS-CoV-2 surrogate virus neutralization tests on deer serum samples by using the Genscript cPass SARS-CoV-2 sVNT Kit (Genescript, https://www.genscript.com)

according to the manufacturer's instructions. Fallow deer serum samples were thawed and heat inactivated at 56°C for 30 minutes in a water bath. Samples and controls were diluted 1:10 and mixed with horseradish peroxidase–labeled receptor-binding domain solution before incubating at 37°C for 30 minutes. Reaction mixtures were added to an ACE2-coated microtiter plate and incubated at 37°C for 15 minutes. The plate was washed and 3,3′,5,5′-tetramethylbenzidine substrate was added to each well, and the plate was incubated in the dark at 25°C for 15 minutes. The reactions were then quenched with stop solution before immediately reading the samples on a Clariostar plate reader (BMG Labtech, https://www.bmglabtech.com).

Nucleic Acid Extraction and SARS-CoV-2 qRT-PCR

Twenty-five mg of each tissue sample was added to 1 mL of TRIzol with a single 5-mm stainless steel bead (QIAGEN, https://www.qiagen.com) and homogenized at maximum speed for 2 minutes by using a TissueLyser II (QIAGEN). Then, 0.1 mL of 1-bromo-3-chloropropane was added to each homogenized sample and shaken vigorously before incubating for 3 minutes. The sample was spun in a precooled centrifuge (4°C, 12,000 × g for 15 minutes), after which 0.45 mL of the upper aqueous layer was transferred to a new tube and combined with 1 equivalent volume of 70% ethanol and mixed gently. The sample was added to RNeasy spin columns (QIAGEN) and the manufacturer's protocol was then followed.

To prepare RNA from cecal content, 1 part cecal content was added to 9 parts 50 mmol/L Tris-HCl (pH 8.0), clarified by centrifugation (4°C, 4000 × g), and filtered through 0.45 µm and 0.22 µm filters. Filtrate was concentrated by using 100 kDa Amicon spin columns (Merck-Millipore, https://www.merckmillipore.com). Free nucleic acids were digested by using OmniCleave Endonuclease (LGC Biosearch Technologies, https://www.biosearchtech.com) for 30 minutes. The RNeasy tissue extraction protocol (QIAGEN) was then followed after the homogenization step.

Generation of Fallow Deer Precision Cut Lung Slices and Tracheal Explants Cultured as Air-Liquid Interface

Lungs from 2 seronegative fallow deer were removed immediately after culling. The tissues underwent gross and microscopic evaluation; no morphologic evidence of respiratory disease was observed in tissues used for ex vivo infections. One lung from each animal was clamped across the mainstem bronchus by using a hemostat and transported to the laboratory on

ice. Each lung was perfused with 2% low-melting temperature agarose (Sigma-Aldrich, https://www.sigmaaldrich.com) via the mainstem bronchus and allowed to set for ≈10 minutes at room temperature. A 2-cm × 2-cm × 1-cm (length × width × height) section of agarose infused lung was dissected and embedded in an agarose mold and cut into 250-µm slices by using a Leica VT1000S vibratome (Leica Biosystems, https://www.leicabiosystems.com). Precision cut lung slices with identical diameters were then generated by using an 8-mm biopsy punch. Slices were transferred to individual wells of a 24-well plate and cultured in 2 mL lung slice medium as previously described (*8*). Tracheal tissue from 2 seronegative animals was removed from the upper third of the trachea at the time of culling, transported to the laboratory on ice, resected, and then cut longitudinally through the trachealis muscle. The mucosa was carefully dissected away from the cartilage and an 8-mm biopsy punch was used to generate tracheal explants. Tracheal explants were cultured on the apical surface of Corning Transwell polycarbonate inserts with 0.4-µm pores (Corning, https://www.corning.com), and 2 mL culture medium was added to the basolateral side of the models creating an air-liquid interface. Tracheal explants were cultured in tracheal explant medium consisting of LHC medium supplemented with 5% heat-inactivated fetal calf serum, 50 U/mL penicillin/streptomycin, 1.25 µg/mL amphotericin B, and 1× Insulin-Transferrin-Selenium solution (GIBCO, Thermo Fisher Scientific, https://www.thermofisher.com).

Immunohistochemistry to Detect SARS-CoV-2 Antigen in Deer Tissue

Formalin-fixed, paraffin-embedded fallow deer trachea and lung sections were immunohistochemically stained to evaluate the expression profiles of SARS-CoV-2 proteins. Consecutive sections were cut at 4-µm thickness. The mouse monoclonal IgG1 1A9 (GeneTex, https://www.genetex.com) directed against SARS-CoV-2 spike protein was used as the primary antibody. Staining was performed by using the DAKO Link 48 Autostainer according to the manufacturer's instructions and the Dako EnVision Flex kit (both Agilent Technologies Inc., https://www.agilent.com). Antigen retrieval was performed by using DAKO PTLink for 20 minutes at 97°C in target retrieval buffer, pH 6 (Agilent Technologies Inc.). Tissue sections were blocked for endogenous peroxidase and nonspecific binding by incubating in 30% hydrogen peroxide (Sigma Aldrich) for 30 minutes and in protein block (Agilent Technologies Inc.) and T20 buffer (Thermo Fisher Scientific) for 15 minutes each. Optimal dilution of SARS-CoV-2 antibody was determined by using a formalin-fixed, paraffin-embedded pellet of Vero E6 cells

infected with SARS-CoV-2 Italy-INMI-1 as a positive control and uninfected cell pellet as a negative control. The optimal antibody dilution was 1:1000 and incubation time was 30 minutes. Tissue sections were then washed and incubated with horse radish peroxidase from the EnVision Flex kit for 20 minutes. The chromogen 3,39-diaminobenzidine was used to visualize positive antibody staining (sections were incubated twice for 5 minutes each). Negative controls were run under identical conditions for each case by replacing the primary antibody with diluent. An isotype control (IHC universal negative control reagent; Enzo Life Sciences, https://www.enzo.com) was also included for each sample. Slides were counterstained with hematoxylin (Agilent Technologies Inc.) and rinsed in deionized water. Slides were dehydrated, permanently mounted, and then scanned and digitized by using the Aperio AT2 Digital Slide Scanner, and images were reviewed by using Aperio ImageScope 12.4 software (both Leica Biosystems).

References

1. Griffin LL, Haigh A, Amin B, Faull J, Norman A, Ciuti S. Artificial selection in human–wildlife feeding interactions. J Anim Ecol. 2022;91:1892–905. PubMed https://doi.org/10.1111/1365-2656.13771

2. Bates D, Mächler M, Bolker B, Walker S. Fitting linear mixed-effects models using lme4. J Stat Softw. 2015;67:1–48. https://doi.org/10.18637/jss.v067.i01

3. Dormann CF, Elith J, Bacher S, Buchmann C, Carl G, Carré G, et al. Collinearity: a review of methods to deal with it and a simulation study evaluating their performance. Ecography. 2013;36:27–46. https://doi.org/10.1111/j.1600-0587.2012.07348.x

4. Robinson GK. That BLUP is a good thing: the estimation of random effects. Statist Sci. 1991;6:15–32. https://doi.org/10.1214/ss/1177011926

5. Stoffel MA, Nakagawa S, Schielzeth H. rptR: repeatability estimation and variance decomposition by generalized linear mixed-effects models. Methods Ecol Evol. 2017;8:1639–44. https://doi.org/10.1111/2041-210X.12797

6. Hadfield JD, Wilson AJ, Garant D, Sheldon BC, Kruuk LEB. The misuse of BLUP in ecology and evolution. Am Nat. 2010;175:116–25. PubMed https://doi.org/10.1086/648604

7. Houslay TM, Wilson AJ. Avoiding the misuse of BLUP in behavioural ecology. Behav Ecol. 2017;28:948–52. PubMed https://doi.org/10.1093/beheco/arx023

8. Cousens C, Alleaume C, Bijsmans E, Martineau HM, Finlayson J, Dagleish MP, et al. Jaagsiekte sheep retrovirus infection of lung slice cultures. Retrovirology. 2015;12:31. PubMed https://doi.org/10.1186/s12977-015-0157-5

9. Corman VM, Landt O, Kaiser M, Molenkamp R, Meijer A, Chu DK, et al. Detection of 2019 novel coronavirus (2019-nCoV) by real-time RT-PCR. Euro Surveill. 2020;25:2000045. PubMed https://doi.org/10.2807/1560-7917.ES.2020.25.3.2000045

Appendix 1 Table. SARS-CoV-2 qRT-PCR primers, probe, and cycling conditions for PCR of the SARS-CoV-2 envelope gene*

Primer name	Primer sequence, 5′–3′	PCR cycling conditions
E_Sarbeco_F	ACAGGTACGTTAATAGTTAATAGCGT	50°C for 300 s, 95°C for 20 s, then 45 cycles of 95°C for 15 s, 59°C for 60 s
E_Sarbeco_R	ATATTGCAGCAGTACGCACACA	
E_Sarbeco_P1	FAM-ACACTAGCCATCCTTACTGCGCTTCG-BBQ	

*Primer and probe names and sequences were obtained from (*9*). BBQ, blackberry quencher; FAM, 6-carboxyfluorescein; F, forward primer; P1, probe 1; qRT-PCR, quantitative reverse transcription PCR; R, reverse primer.
